# The Integrity of piRNA Clusters is Abolished by Insulators in the *Drosophila* Germline

**DOI:** 10.3390/genes10030209

**Published:** 2019-03-11

**Authors:** Elizaveta Radion, Olesya Sokolova, Sergei Ryazansky, Pavel A. Komarov, Yuri Abramov, Alla Kalmykova

**Affiliations:** 1 Institute of Molecular Genetics, Russian Academy of Sciences, 123182 Moscow, Russia; sradion-radion.90@mail.ru (E.R.); sokolova@img.ras.ru (O.S.); ryazansky@img.ras.ru (S.R.); pkom94@gmail.com (P.A.K.); abramov75@rambler.ru (Y.A.); 2 Present Address: Friedrich Miescher Institute for Biomedical Research, Maulbeerstrasse 66, 4058 Basel, Switzerland

**Keywords:** drosophila, retrotransposons, transgene, piRNA cluster, insulator, Su(Hw), Rhino, germline, transcription, *HeT-A* and *TART* telomeric retrotransposons

## Abstract

Piwi-interacting RNAs (piRNAs) control transposable element (TE) activity in the germline. piRNAs are produced from single-stranded precursors transcribed from distinct genomic loci, enriched by TE fragments and termed piRNA clusters. The specific chromatin organization and transcriptional regulation of *Drosophila* germline-specific piRNA clusters ensure transcription and processing of piRNA precursors. TEs harbour various regulatory elements that could affect piRNA cluster integrity. One of such elements is the suppressor-of-hairy-wing (Su(Hw))-mediated insulator, which is harboured in the retrotransposon *gypsy*. To understand how insulators contribute to piRNA cluster activity, we studied the effects of transgenes containing *gypsy* insulators on local organization of endogenous piRNA clusters. We show that transgene insertions interfere with piRNA precursor transcription, small RNA production and the formation of piRNA cluster-specific chromatin, a hallmark of which is Rhino, the germline homolog of the heterochromatin protein 1 (HP1). The mutations of Su(Hw) restored the integrity of piRNA clusters in transgenic strains. Surprisingly, Su(Hw) depletion enhanced the production of piRNAs by the domesticated telomeric retrotransposon *TART*, indicating that Su(Hw)-dependent elements protect *TART* transcripts from piRNA processing machinery in telomeres. A genome-wide analysis revealed that Su(Hw)-binding sites are depleted in endogenous germline piRNA clusters, suggesting that their functional integrity is under strict evolutionary constraints.

## 1. Introduction

The Piwi-interacting RNA (piRNA) pathway is an essential mechanism that protects genome integrity by suppressing transposable element (TE) activity in animal gonads [1]. In *Drosophila*, piRNA precursors are derived from distinct genomic regions termed piRNA clusters, which are enriched in TE fragments [2]. The specific chromatin structure of piRNA clusters ensures the recruitment of the noncanonical transcriptional machinery that drives piRNA precursor expression [3,4,5,6]. The chromatin of piRNA clusters is enriched in a common heterochromatic histone mark, trimethylated lysine 9 of histone H3 (H3K9me3) and by two chromodomain-containing proteins, heterochromatic protein 1 (HP1) and its germline-specific ortholog Rhino (Rhi) [4,7,8,9]. The protein Maelstrom represses canonical transcription from TEs and neighbouring gene promoters in dual-strand piRNA clusters [10]. Instead, noncanonical convergent transcription from both genomic strands initiated at multiple random sites facilitates the transcription of piRNA precursors from dual strand piRNA clusters [6,7]. The initiation of such noncanonical transcription within the heterochromatin of piRNA clusters is mediated by the germline-specific TFIIA-L paralog Moonshiner, which forms an alternative RNA Polymerase II preinitiation complex in Rhi-enriched domains [6]. Rhi binding suppresses the splicing of piRNA precursors [11]. In addition, Rhi recruits Cutoff (Cuff) protein [4], which mediates the generation of long read-through transcripts from piRNA clusters by inhibiting termination at poly(A) sites [12]. Finally, the transcription-export (TREX) complex participates in the export of unspliced piRNA precursors from the nucleus to the cytoplasmic piRNA processing machinery [13,14]. The Piwi-dependent establishment of piRNA cluster identity occurs during early embryogenesis, which is crucial for TE repression at later developmental stages [9]. The integrity of piRNA clusters is an important factor in antitransposon defence, since the adaptivity of the piRNA system is based on the ability of alien sequences inserted within piRNA clusters to become their integral part and produce cognate piRNAs [15,16,17,18].

Insulators and their binding proteins play an essential role in transcription regulation by limiting inappropriate enhancer-promoter interactions of neighbouring genes or by blocking repressive chromatin spreading [19]. Insulators are found in the regulatory regions of genes and at homeotic gene loci and the boundaries of topologically associating domains, TADs [20,21]. Some *Drosophila* retrotransposons also contain insulators [22,23,24]. One of the best-characterized TE insulators is located in the regulatory region of the *gypsy* long terminal repeat (LTR) retrotransposon and contains binding sites for the suppressor of hairy wing (Su(Hw)) zinc-finger protein [25]. This DNA-binding protein establishes the multicomponent chromatin complex important for transcriptional regulation and germline development [19,26]. piRNA clusters contain different TEs, including those demonstrating insulator activity; however, the hierarchical relationship between the chromatin of piRNA clusters and insulator complex formation is not clear. We show here that transgenes bearing Su(Hw) recognition sites embedded in endogenous pericentromeric and telomeric piRNA clusters interfere with the local transcription of piRNA precursors, production of small RNAs and formation of specific chromatin structure.

## 2. Materials and Methods

### 2.1. Drosophila Transgenic Strains

The transgenic strain *KG10047* carrying the insertion of the P{SUPor-P} element in the *HeT-A* 3′ UTR was described previously [27]. Transgenic strain *KG09351* (Bloomington Drosophila Stock Centre #16481; the strain was terminated) carries a P{SUPor-P} insertion in the *42AB* locus at the position 2R:2,160,357 [-] (according to the dm3 genome assembly). The transgenic strain KG02245 (Bloomington Drosophila Stock Centre #12975) carries a P{SUPor-P} insertion in the *49E* locus. Transheterozygous *su(Hw)^V^/su(Hw)^f^* flies were used in the study.

### 2.2. Small RNA Library Preparation and Analysis

Small RNAs 19–29 nt in size from total ovarian RNA extracts were cloned as previously described [18]. The libraries were barcoded according to Illumina TrueSeq Small RNA sample prep kit instructions and submitted for sequencing using the Illumina HiSeq-2000 sequencing system (San Diego, CA, USA). After clipping the Illumina 3′-adapter sequence, small RNA reads that passed quality control and minimal length filter (>18 nt) were mapped (allowing 0 mismatches) to the Drosophila melanogaster genome (Apr. 2006, BDGP assembly R5/dm3) or transgenes by bowtie2 [28]. The plotting of size distributions, read coverage and nucleotide biases were performed as described previously [18]. To identify piRNAs (24–29 nt reads) or siRNAs (21 nt reads) derived from TEs and piRNA clusters, small RNA reads were mapped to the canonical sequences of transposable elements (http://www.fruitfly.org/p_disrupt/TE.html) or to the piRNA clusters [2] by bowtie2 [28].

Ovarian small RNA-seq data for *KG10047*;+/+, *KG10047*;*su(Hw)^V^/su(Hw)^f^*, *KG09351*;+/+; *KG09351*;*su(Hw)^V^/su(Hw)^f^* ; KG02245;+/+ and KG02245;*su(Hw)^V^/su(Hw)^f^* were deposited at Gene Expression Omnibus (GEO), accession number GSE125173.

### 2.3. Chromatin Immunoprecipitation (ChIP)

ChIP was performed according to the published procedure [7]. Chromatin was immunoprecipitated with the following antibodies: anti-HP1a (C1A9 Developmental Studies Hybridoma Bank, Iowa Sity, IA, USA), anti-trimethyl-histone H3 Lys9 (Millipore, Burlington, MA, USA), Rhi antiserum [29] and anti-Su(Hw) [30]. Primers used in the study are listed in Table S1. Quantitative PCR was conducted with a LightCycler 96 (Roche, Basel, Switzerland). Obtained values were normalized to input and compared with values at *rp49* gene as a control genomic region. Standard error of mean (SEM) of triplicate PCR measurements for three biological replicates was calculated.

### 2.4. RT-PCR

RNA was isolated from the ovaries of three-day-old females. cDNA was synthesized using random hexamers or strand-specific primers and SuperScriptII reverse transcriptase (Life Technologies, Carlsbad, CA, USA). cDNA samples were analysed by real-time quantitative PCR using SYTO-13 dye on a LightCycler96 (Roche, Basel, Switzerland). Values were averaged and normalized to the expression level of the ribosomal protein gene *rp49*. The primers used are described in Table S1.

### 2.5. Motif Finding

To estimate the frequency of the Su(Hw) insulator sites, the corresponding PWM profile (MA0533.1 from JASPAR_2016 database) was searched against the dm6 genome assembly or piRNA cluster regions [2] by using fimo 4.11.1 from the MEME suite [31]. The *p*-value 1 × 10^−5^ was used as the threshold level.

## 3. Results and Discussion

### 3.1. P{SUPor-P} Transgenic Constructs Inserted into piRNA Clusters Do Not Produce piRNAs

**Figure 1:**
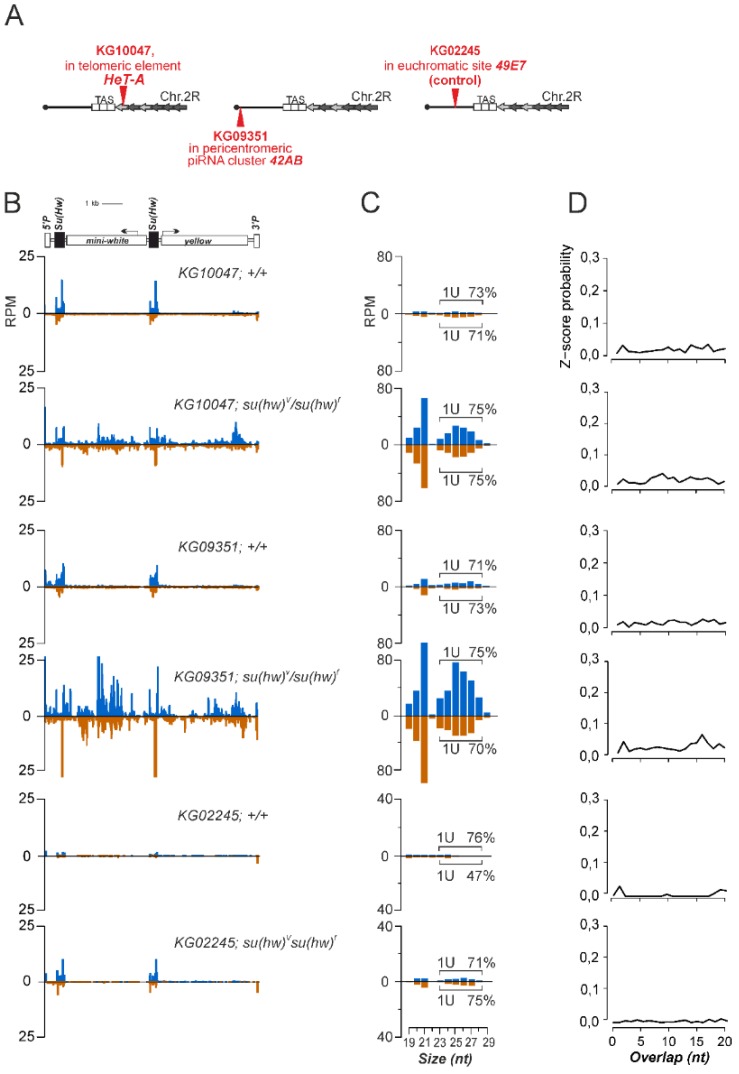
Generation of small RNAs by transgenes containing Su(Hw)-binding sites and located in piRNA clusters. (**A**) Schematic representation of transgenic insertion sites. Insertion sites of transgenes are indicated as triangles situated either up or below the schemes, which correspond to their genomic orientation. (**B**) Scheme of SUPor-P construct is shown above. Normalized numbers of small RNAs (19–29 nt, in reads per million, RPM) mapped to transgenic constructs (blue—sense; brown—antisense; no mismatches allowed) in wild type *Drosophila* ovaries and in *su(Hw)^V^/su(Hw)^f^* mutants. (**C**) Length distribution of transgenic small RNAs. Percentage of transgenic reads excluded Su(Hw) sites having 1U is indicated for each strand (only 24–29-nt reads were considered). (**D**) Relative frequencies (Z-score) of 5′ overlap for sense and antisense 24–29-nt transgenic piRNAs excluded Su(Hw) sites (ping-pong signature).

To study how insulators affect piRNA cluster integrity, we used P{SUPor-P} transgenic constructs carrying two *gypsy* insulators and located within endogenous piRNA clusters. KG10047 and KG09351 transgenes were inserted into the 3′UTR of telomeric retrotransposon *HeT-A* and the major pericentromeric piRNA cluster in the *42AB* locus, respectively (Figure 1A).

**Figure 2:**
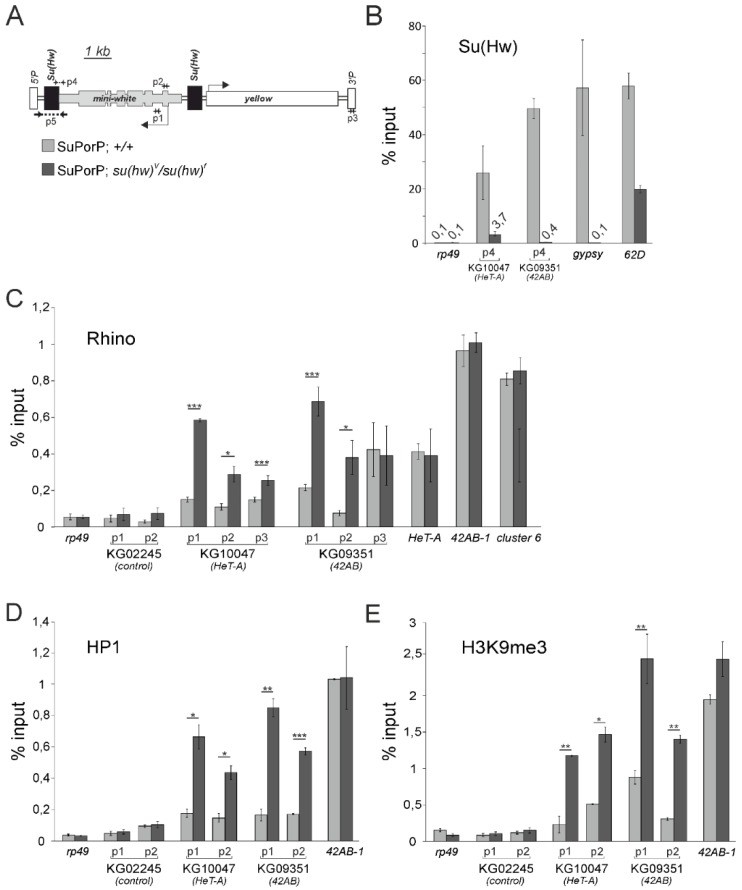
Chromatin components and transcription state of the transgenes containing Su(Hw) sites and located within piRNA clusters. (**A**) Schematic representation of the P{SUPor-P} transgene and the positions of the primers used in ChIP (p1, p2, p3, p4) and RT-PCR (p1, p3, p5) are shown. (**B**) Su(Hw) binds P{SUPor-P} transgenes in Su(Hw) wild type but not in mutant ovaries. The *rp49* is used as a control devoid of Su(Hw) binding sites. As expected, the *gypsy* insulator lost Su(Hw) binding in the *su(Hw)^V^/su(Hw)^f^* background but the insulator in the *62D* locus retained Su(Hw) association [33,34]. Mean values are indicated only for low levels of Su(Hw) binding. (**C**–**E**) Rhi (**C**), HP1 (**D**) and H3K9me3 (**E**) occupancies at P{SUPor-P} transgenes in wild type and *su(Hw)^V^/su(Hw)^f^* mutants were estimated by ChIP-qPCR using indicated primers (p1, p2, p3). The regions of the endogenous *42AB* and #6 piRNA clusters and telomeric retrotransposon *HeT-A* are enriched by all studied chromatin components and used as positive controls. *rp49* is used as a negative control. Asterisks indicate statistically significant differences in chromatin protein enrichments between wild type and *su(Hw)^V^/su(Hw)^f^* mutants (* *p* < 0.05 to 0.01, ** *p* < 0.01 to 0.001, *** *p* < 0.001, unpaired *t*-test). Error bars represent SEM of 3 biological replicate experiments. For HP1 and H3K9me3 binding to KG02245, the error bars represent SD of three technical replicates.

Both integrated loci were previously described as potent piRNA clusters able to adapt new insertions for piRNA production [12,15,16,32]. The euchromatic KG02245 transgene was used as a control. To determine whether the constructs carrying *gypsy* insulators are able to become a part of piRNA clusters and produce piRNAs, we sequenced small RNAs from the ovaries of the transgenic strains. The mapping of small RNAs to P{SUPor-P} revealed a negligible amount of the transgene-derived small RNAs in both cases (Figure 1B). We suggested that Su(Hw) binding could impede piRNA production and performed ovarian small RNA sequencing of transgenic strains bearing *su(Hw)^V^/su(Hw)^f^* mutations. These mutations cause the loss of Su(Hw) binding to the *gypsy* insulator [33,34] and, as we show in the next section, to P{SUPor-P} transgene (Figure 2B). Su(Hw) mutations result in the production of abundant transgenic small RNAs, most of which are 24–29 nt long and demonstrate 5′ terminal uridine bias (1U bias), which is a characteristic of piRNAs (Figure 1B,C). However, we did not find the sense/antisense piRNA pairs overlapping by 10 nt, which is a signature of the ping-pong piRNA amplification cycle [2,35] (Figure 1D). This result indicates that primary processing plays a major role in transgenic piRNA production. The most likely explanation is that a low abundance of transgenic transcripts prevents efficient ping-pong amplification. The euchromatic transgene KG02245 (control) produces a negligible amount of small RNAs in wild type and Su(Hw) mutant backgrounds (Figure 1).

Interestingly, a significant fraction of the small RNAs produced by both P{SUPor-P} transgenes in Su(Hw) mutants are 21-nt endogenous small interfering RNAs (endo-siRNAs) (Figure 1C). Indeed, it has been reported that endogenous and transgenic piRNA clusters also produce significant levels of endo-siRNAs in wild type ovaries [18,32,36,37]. In Su(Hw) mutants, P{SUPor-P} transgenes become part of the endogenous piRNA clusters, producing both pi- and endo-siRNAs.

Previously, it was reported that P{lArB}, pW8-hsp-pA and P{EPgy2} transgenes lacking *gypsy* insulators inserted in *Drosophila* subtelomeric and telomeric piRNA clusters are incorporated in piRNA production and acquire chromatin properties of their surrounding regions [16,18,32]. Similar to transgenes, TE insertions into the germline piRNA clusters result in the production of cognate piRNAs ensuring silencing of mobilized TEs [15]. We show here, that Su(Hw)-mediated *gypsy* insulators prevent piRNA production from P{SUPor-P} transgenic sequences, even if the latter are inserted into endogenous piRNA-producing regions.

### 3.2. The Su(Hw) Complex Prevents the Assembly of the Chromatin Structure and Read-Through Transcription Typical of piRNA Clusters

To learn more about the mechanism of the Su(Hw)-mediated prevention of piRNA production, we compared the chromatin state of transgenes in the wild type and Su(Hw)-mutant backgrounds. In our experiments, we used transheterozygous flies with two Su(Hw) alleles: the Su(Hw)^V^ null allele and the Su(Hw)^f^, which carry a defective zinc finger 10 [34]. Su(Hw)^f^ protein demonstrated the loss of binding to *gypsy* insulator and the reduced occupancy of many non-g*ypsy* Su(Hw)-binding sites [34,38]. ChIP data obtained using anti-Su(Hw) antibodies demonstrate that P{SUPor-P} transgenes binds Su(Hw) in wild type ovaries (Figure 2B). ChIP performed with *su(Hw)^V^/su(Hw)^f^* ovaries, shows a dramatic decrease of Su(Hw) binding to the *gypsy* insulator and P{SUPor-P} transgenes. As it was reported previously, mutant Su(Hw)^f^ is retained at some insulators including site located in *62D* locus [33,34]. Indeed, ChIP demonstrated that mutant Su(Hw) bound this region, that serves as a positive control (Figure 2B).

The data from ChIP using Rhi, HP1 and H3K9me3 antibodies show that P{SUPor-P} transgenes inserted in both the *42AB* locus and telomeric retroelement *HeT-A* lack these chromatin hallmarks in the presence of Su(Hw) protein (in the wild type genetic background) but acquire them in Su(Hw)-mutant ovaries (Figure 2C–E). At the same time, Rhi binding to 3′ P-element region, located ~5 kb apart from the *gypsy* insulators, is not affected by Su(Hw) mutation in *KG09351* and only 1.5-fold increases in *KG10047*;*su(Hw)^V/f^* ovaries (Figure 2C, p3 primer pair). Chromatin of the telomeric element *HeT-A* and dual-strand piRNA clusters (*42AB* and cluster #6) was not affected by Su(Hw) mutation (Figure 2). Therefore, the *gypsy* insulator complex is established upstream of the Piwi-dependent chromatin formation of piRNA clusters and Su(Hw) binding mostly affects local chromatin conformation.

**Figure 3:**
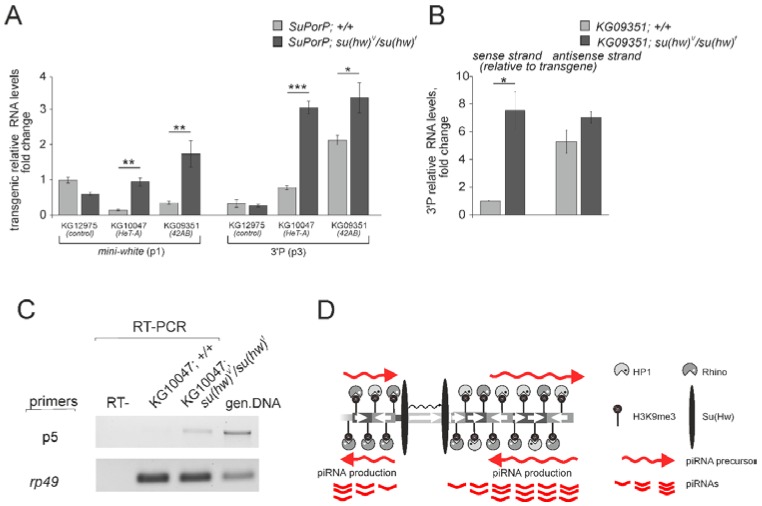
Transcription state of the transgenes containing Su(Hw) sites and located within piRNA clusters. The positions of primers are indicated in Figure 2A. (**A**) RT-qPCR analysis of transgenic *mini-white* and 3′P transcripts in the ovaries of transgenic strains in wild type and Su(Hw) mutant backgrounds. The P1 primer pair used for RT-PCR specifically detects unspliced transgenic *mini-white* transcripts. (**B**) Strand-specific RT-PCR using the P3 primers corresponding to the transgenic 3′P region showed a decreased level of RNA from sense but not from antisense transgenic strand downstream of the Su(Hw) binding sites. Asterisks indicate statistically significant differences in the expression levels between wild type and *su(Hw)^V^/su(Hw)^f^* mutants (* *p* < 0.05 to 0.01, ** *p* < 0.01 to 0.001, *** *p* < 0.001, unpaired *t*-test). (**C**) Agarose gel electrophoresis of RT-PCR products shows the presence of read-through transgenic transcripts comprising Su(Hw)-binding sites only in Su(Hw) mutants. Samples without reverse transcriptase were used as RT^−^ controls. PCR on genomic DNA served as a positive control. (**D**) Scheme showing that insertion of the Su(Hw) insulator into the piRNA cluster disrupts local transcription of piRNA precursors, production of small RNAs and formation of specific chromatin structure.

Our data suggest that Su(Hw) binding to P{SUPor-P} transgenes inserted into piRNA clusters should block transcription of long piRNA precursors. To verify this suggestion, we compared expression of *mini-white* gene of the transgenes inserted into *HeT-A* (*KG10047* strain) and *42AB* (*KG09351* strain) in the wild-type and mutant background using transgene-specific primers (Figure 3A). It should be noted that the *white* promoter shows a very low activity in ovaries. In contrast, we observed that the level of *mini-white* transcripts was significantly increased in Su(Hw) mutants for both insertions (Figure 3A). In addition, Su(Hw) mutations lead to the increased transcript levels of 3′P transgenic regions located 5 kb downstream Su(Hw) binding sites (Figure 3A). Strand-specific RT-PCR analysis of 3′P transcription in *KG09351* strain demonstrated a lowered level of only sense transgenic transcripts suggesting that the effect of *gypsy* insulator on transcription is strand-specific (Figure 3B). We therefore suggest that the Su(Hw)-insulator complex should directly interfere with transcription of piRNA clusters by blocking read-through transcription of piRNA precursors. To test this, we conducted RT-PCR using the primers corresponding to the upstream and downstream regions of the transgenic Su(Hw) site (Figure 2A, p5 primer pair). Accordingly, we revealed transgenic transcripts only in the ovaries of Su(Hw) mutants (Figure 3C). These data indicate that the insulator complex blocks the read-through transcription of transgenic piRNA precursors. Taken together, our data suggest that insertions of the transgenes containing *gypsy* insulators into endogenous piRNA clusters affect local chromatin conformation, causing the disruption of long piRNA precursor transcription and that this effect is mediated by Su(Hw) binding (Figure 3D). However, the function of the *gypsy* insulator also requires Centrosomal Protein 190 kD (CP190) and Modifier of mdg4 (Mod67.2) [39,40], the insulator proteins which were not considered here. Therefore, strictly speaking, we could not unambiguously conclude whether or not the effect of Su(Hw) binding to piRNA clusters demonstrated here was dependent on the entire insulator complex assembly.

Given the functional integrity of piRNA clusters, we suggested that *gypsy* insulators might impair the functioning of the cluster regions in the close vicinity of transgenic insertion. To verify this suggestion, we estimated piRNA production in *42AB* regions located around the KG09351 insertion.

**Figure 4:**
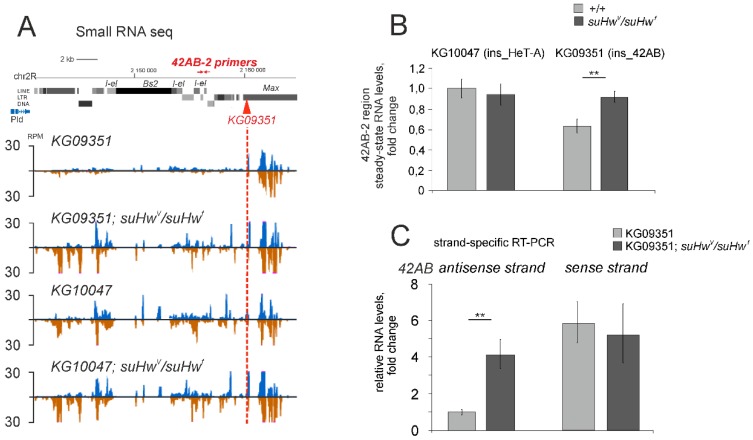
Su(Hw)-binding sites disrupt the integrity of the endogenous piRNA cluster. (**A**) The effect of SUPor-P transgene inserted in the *42AB* locus on piRNA expression. Profile of ovarian small RNA density at the *42AB* region adjacent to the KG09351 transgene in wild type and *su(Hw)^V^/su(Hw)^f^* mutant flies and in the *KG10047* strain without a transgene insertion in this region. The position of the transgene in the minus genomic strand is designated by a red rectangle. Genomic coordinates are indicated according to the dm6 genome assembly. (**B**) RT-qPCR analysis of the expression levels of the *42AB* region located 4 kb downstream of the KG09351 transgene. The positions of the primers used for RT-PCR are schematically indicated in (**A**). (**C**) Strand-specific RT-PCR using the primers indicated in (**A**) showed a decreased level of RNA from the negative genomic strand downstream of the transgene insertion. Asterisks indicate statistically significant differences in the expression levels between wild type and *su(Hw)^V^/su(Hw)^f^* mutants (* *p* < 0.05 to 0.01, ** *p* < 0.01 to 0.001, *** *p* < 0.001, unpaired *t*-test).

Due to the presence of highly degenerated TE fragments, piRNA clusters produce abundant piRNAs uniquely mapped to the genome, allowing their mapping to repeat-rich regions [2]. We found that the amount of piRNAs uniquely mapped to the 10-kb region flanking the 3′-end of the transgene was dramatically lower in the presence of the transgene insertion than in the native *42AB* locus (Figure 4A).

However, Su(Hw) mutations restored the level of transgene-flanking piRNAs in the *42AB* locus up to the level observed in the native *42AB* region in *KG10047* strain (Figure 4A). We also show that the production of piRNAs derived from the same region is not affected by Su(Hw) mutations in *KG10047* strain. In addition, the transgenic insertion in *42AB* leads to a reduction in transcript levels downstream of the transgene (Figure 4B, transgene is located in the minus genomic strand). Strand-specific RT-PCR analysis using single-mapped primers [41] demonstrated a dramatic decrease in the level of transcripts from the negative genomic strand downstream the transgene (Figure 4C). This fact is in agreement with previous result, demonstrating the lowered levels of transgenic 3′P sense transcripts (corresponding to the minus genomic strand) in the ovaries of *KG09351* strain (Figure 3B). Thus, the Su(Hw) insulator blocks piRNA precursor transcription at least within a 10-kb neighbouring region. Our observations suggest that transgenes containing Su(Hw)-binding sites disrupt long transcription units within piRNA clusters and that this effect is strand-specific. Surprisingly, transgene insertion resulting in lowered level of only antisense transcripts led to the decreased level of both sense and antisense piRNAs uniquely mapped to transgene flanking region in *42AB* (Figure 4A). This fact can be explained by an impaired efficiency of ping-pong amplification between piRNA precursors derived from this region. Indeed, it was reported that sense and antisense transcripts originated from the heterochromatic piRNA cluster are involved in the reciprocal cleavage in the course of ping-pong piRNA amplification [42]. Nevertheless, the total abundance of *I*-specific piRNAs is not affected by the KG09351 insertion in *42AB* (Figure S1) because numerous active *I*-element copies participate in piRNA production in this strain.

Taken together, these data explain why Su(Hw) binding results in a local decrease in small RNA production not only from the *mini-white* located between the insulator sequences but also from the transgenic and genomic flanking regions.

We could not perform the analysis of flanking piRNAs near the KG10047 transgene inserted in the *HeT-A* 3′UTR at 2R telomere because the unique mapping of small RNAs to poorly assembled and highly repetitive telomeric regions was technically impossible.

To a certain extent, insertion of P{SUPor-P} transgene in *42AB* was helpful for understanding of the transcription regulation of the major uni-strand *flamenco* (*flam*) locus that controls TE expression in ovarian follicular cells [43,44]. In contrast to the germline dual-strand piRNA clusters that generate piRNAs corresponding to both genomic strands, the *flam* locus produces primary piRNAs from single strand precursors [2]. Insulator-harbouring TEs, such as *gypsy*, *ZAM* and *Idefix* [22,23,24], are exceptionally arranged in antisense orientation relative to *flam* transcription. It is believed that piRNAs complementary to the coding transcripts of these TEs are produced from the single strand precursors transcribed by the *flam* sense strand [2]. Apparently, *gypsy*, *Zam* and *Idefix* insulators do not interfere with the transcription of the *flam* piRNA precursors, likely due to a strand-specific mode of insulator influence on transcription.

### 3.3. Su(Hw) Restricts piRNA Production from Telomeric TART Retrotransposons

The main structural telomeric element, *HeT-A,* is a non-autonomous retroelement and reverse transcriptase (RT) activity is likely to be provided by *TART* or *TAHRE* telomeric retrotransposons. In *Drosophila* germline, telomeric regions are organized in the piRNA clusters, although telomeric elements are heterogeneous in piRNA production and Rhi binding: *TART* retrotransposons are less susceptible to piRNA production and Rhi deposition than *HeT-A* [32]. This implies that *TART* transcripts may be protected by an unknown mechanism from piRNA biogenesis machinery to provide stable expression of RT essential for telomere elongation in the germline. Here, we present the data suggesting a role for Su(Hw) in this mechanism.

**Figure 5:**
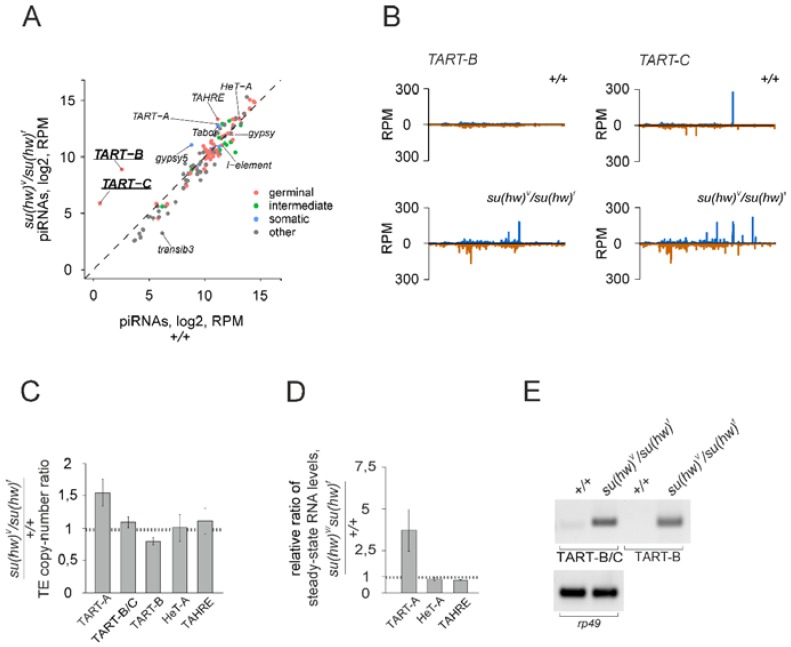
Su(Hw) depletion leads to the increased production of *TART* piRNAs. (**A**) Scatter plots of log2-transformed and RPM-normalized small RNAseq reads in the ovaries of wild type and *su(Hw)* mutant flies mapped to the canonical TE sequences. The colour of the dots indicates the type of TEs according to their capacity for maternal deposition in embryos according to [45]. (**B**) Small RNA mapping to canonical *TART-B* and *TART-C* telomeric retrotransposons. Reads mapped to the sense strand are shown in blue and antisense in brown. Analysis of ovarian small RNA libraries from *KG10047*;+/+ and *KG10047*;*su(Hw)^V^/su(Hw)^f^* strains (0–3 mismatches allowed) is shown (**A**,**B**). (**C**) qPCR on the genomic DNA was done to estimate relative copy number of telomeric retrotransposons in *KG10047;su(Hw)^V^/su(Hw)^f^* compared to *KG10047*;+/+ strain. Normalization to the single-copy *rp49* gene was done. (**D**) RT-qPCR analysis of transcript levels of *TART-A, HeT-A* and *TAHRE* telomeric elements normalized to *rp49* in the ovaries. Shown are fold changes of steady-state RNA levels in KG10047; *su(Hw)^V^/su(Hw)^f^* compared to *KG10047*;+/+ strain. (**E**) Agarose gel electrophoresis of RT-PCR products demonstrates increased levels of *TART-B* and *TART-C* transcripts in Su(Hw) mutants. *Rp49* is used as a loading control.

Using small RNAseq data, we studied the genome-wide impact of Su(Hw) depletion on transposon-derived piRNA production. The mapping of small RNAs to a canonical set of TEs does not demonstrate global changes caused by Su(Hw) mutations (Figure 5A, Figure S2).

However, piRNA production from few TEs was strongly affected (Table S2). In particular, Su(Hw) mutations caused a 100-fold increase in the abundance of piRNAs specific to *TART-B* and *TART-C* subfamilies of telomeric retrotransposons (Figure 5B, Table S2). To exclude the influence of copy number polymorphism, we evaluated the relative copy number of telomeric retrotransposons in *KG10047*;+/+ and *KG10047*;*su(Hw)^V^/su(Hw)^f^* strains. To this end, we performed PCR on genomic DNA and showed that the relative copy numbers of *HeT-A, TAHRE, TART-A, TART-B* and *TART-C* are very similar in both strains (Figure 5C). These data strongly suggest that *TART-B/TART-C* transcripts are protected by Su(Hw)-dependent border elements from the piRNA production. Next, we examined the RNA levels of telomeric retroelements in the ovaries of Su(Hw) mutants by RT-qPCR. We found an increased level of *TART-A* transcripts in the ovaries of Su(Hw) mutants, while *HeT-A* and *TAHRE* expression was not affected (Figure 5D). However, the levels of *TART-B* and *TART-C* transcripts in the wild type ovaries were undetectable by RT-qPCR. Probably, *TART* expression is limited by a time window during oogenesis, resulting in low levels of *TART* RNA in the total ovarian RNA. Then, we performed semiquantitative RT-PCR and observed that *TART-B* and *TART-C* transcripts were barely revealed in the wild type ovaries but readily detected in the ovaries of Su(Hw) mutants (Figure 5E). These data suggest that Su(Hw)-dependent insulators provide appropriate levels of *TART*-encoding transcripts in telomeres. Su(Hw) serves as a transcriptional repressor of coding genes in the ovary [26]. Notably, in wild-type ovaries, *TART* transcripts are less abundant than upon Su(Hw) depletion. Thus, Su(Hw) likely mediates transcriptional repression of *TART* in the germline.

### 3.4. Su(Hw)-Binding Sites Are Depleted from Dual-Strand piRNA Clusters

What could be happened if the insulator is inserted into the piRNA cluster? To some extent, this situation is simulated by the insertion of P{SUPor-P} transgene into the *42AB* cluster (Figure 4). Interestingly, the region of the KG09351 insertion in *42AB* harbours remnants of ancestral *I*-related retrotransposon, producing abundant piRNAs playing a key role in the control of *I*-element activity [41,46]. The integration of insulator-containing TE nearby *I*-element fragments in *42AB* would strongly decrease the abundance of piRNAs derived from this region and the resistance to *I*-element mobilization in the strain. One may suggest that shaping endogenous piRNA clusters should be under the constraint of adaptive evolution and, therefore, the germline-specific piRNA clusters should be depleted of insulator-binding sites. To examine this idea, we estimated the average density of Su(Hw)-binding sites in the whole genome and in piRNA clusters (see Materials and Methods). We found that the Su(Hw) site density was lower in piRNA clusters (one site per 45 kb) than in the genome (one site per 14 kb, *p*-value < 1 × 10^−5^). It is tempting to speculate that the spectrum of regulatory sequences associated with TEs in piRNA clusters is subjected to strict selection to provide functional integrity of piRNA-producing loci.

**Figure 6:**
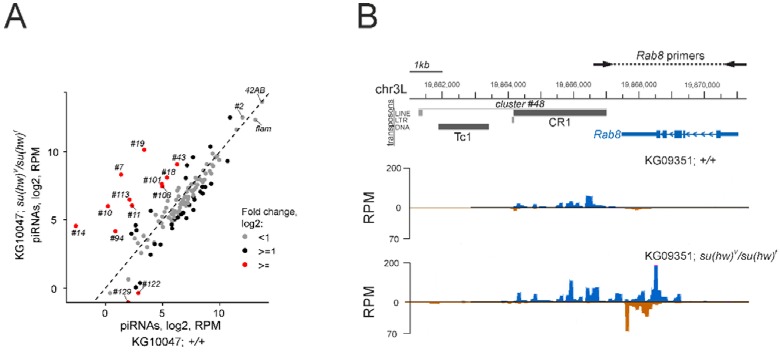
Su(Hw) depletion leads to increased production of piRNAs in distinct genomic sites. (**A**) Scatter plot of log2-transformed and RPM-normalized small RNAseq reads in the ovaries of wild type and *su(Hw)^V^/su(Hw)^f^* mutant flies mapped to piRNA clusters (**B**) Profile of the ovarian small RNA density at the *Rab8* gene region located in close proximity to cluster #48, comprising of *Tc1* and *CR1* TEs, in wild type and *su(Hw)^V^/su(Hw)^f^* mutant flies. The increased production of small RNAs by both genomic strands in the Su(Hw) mutant background is shown. Small RNAseq data from the ovaries of *KG10047*;+/+ and *KG10047*;*su(Hw)^V^/su(Hw)^f^* strains were used in the figure. Genomic coordinates are indicated according to the dm6 genome assembly. PCR on genomic DNA using indicated primers did not reveal variations in the length of the *Rab8* gene region.

Indeed, mapping of small RNAseq reads to the annotated piRNA clusters [2] did not reveal global changes in the abundance of pi- and endo-siRNAs caused by Su(Hw) mutations (Figure 6A, Figure S3). Of those affected are many piRNA clusters related to telomeric regions which contain *TART* and *TAHRE* retrotransposons (Table S2). However, motif search analyses with the same parameters as above (*p*-value < 1 × 10^−5^) failed to identify the Su(Hw) binding sites in canonical copies of telomeric retrotransposons. Thus, the nature of Su(Hw) binding sites in telomeres is still to be determined.

### 3.5. The Su(Hw) Complex Protects Coding Genes from Spurious piRNA Production in the Germline

Although the loss of Su(Hw) causes female sterility, its particular role in the female germline development is not well understood [34,38]. Su(Hw) mutations lead to the increased expression of many target genes in the ovary, suggesting that Su(Hw) serves as a transcriptional repressor during oogenesis [26].

The genome-wide analysis of small RNAseq data demonstrated additional functions of Su(Hw) in the germline. We revealed a genome-wide effect of *Su(Hw)^V^/Su(Hw)^f^* mutations on the abundance of piRNAs derived from coding genes (Table S3). Most of the affected genes do not produce piRNAs in a wild-type ovary. The piRNAs corresponding to these genes were observed in *Su(Hw)^V^/Su(Hw)^f^* ovaries, suggesting that Su(Hw) might prevent the spread of piRNA production from piRNA clusters/TEs to neighbouring genes. Indeed, Su(Hw) depletion causes the appearance of piRNAs antisense to the transcripts of the *Rab8* gene located next to the cluster #48 (Figure 6B). PCR by using genomic DNA did not reveal variations in the length of the gene region producing genic piRNAs, rejecting the possibility of transposon insertion polymorphism. Thus, the insulator most likely blocks transcription of long piRNA precursors, thus protecting coding gene transcripts from entering the piRNA biogenesis machinery in the germline. However, annotated TEs were found in the close vicinity of the affected genes only in a few cases. Surprisingly, we revealed an effect of Su(Hw) mutations on the abundance of the piRNAs derived from dozens of the coding genes located far from annotated TEs or piRNA clusters (Table S3). At least partly, genic piRNA production may be explained by strain-specific transposon insertions [47]. In most cases, the molecular mechanisms responsible for generation of genic piRNAs in the ovaries of Su(Hw) mutants remain unclear. It is tempting to speculate that Su(Hw)-mediated complexes perform a barrier function to protect coding gene transcripts from spurious piRNA production in the germline.

## 4. Conclusions

Genomic regions containing damaged transposon fragments were for a long time considered as waste dumps. Most of these regions are piRNA-producing loci that play an essential role in antitransposon defence. The complex regulation of piRNA clusters has evolved to provide piRNA production from the entire piRNA cluster. This mechanism ensures an adaptive response to insertions of alien transposons in a piRNA cluster. We show that Su(Hw) binding sites disrupt the integrity of endogenous piRNA clusters, indicating that the assembly of insulator complexes occurs upstream of cluster-specific chromatin formation. Considering that distinct TE families comprise insulator-binding sites and other regulatory sequences, the TE content of the piRNA clusters should be under strict evolutionary constraints. Moreover, Su(Hw)-mediated complexes likely protect telomeric retrotransposon *TART* and coding gene transcripts from spurious piRNA production in the germline.

## Supplementary Materials

The following are available online at https://www.mdpi.com/2073-4425/10/3/209/s1, Figure S1: Small RNA mapping to the canonical *I*-element. Figure S2: Genome-wide analysis of Su(Hw) depletion on TE piRNA production (related to Figure 5). Figure S3: Genome-wide analysis of Su(Hw) depletion on small RNA production from piRNA clusters and genes (related to Figure 6). Table S1. Primers used in the study (5′-to-3′). Table S2. Su(Hw) mutations affect piRNA production from some TEs and piRNA clusters. Table S3. Su(Hw) mutations cause accumulation of genic piRNAs.
