# Supplementary material for: The Integrity of piRNA Clusters is Abolished by Insulators in the Drosophila Germline
Source: Genes (Basel). 2019 Mar 11;10(3):209. doi: 10.3390/genes10030209 (PMC6471301; doi:10.3390/genes10030209)
Supplement: Supplementary file 1 [file genes-10-00209-s001.zip › Supplementary figures S1-S3.docx]

**Supplementary figures S1-S3**

**The integrity of piRNA clusters is abolished by insulators in the *Drosophila* germline**

Elizaveta Radion^1^, Olesya Sokolova^1^, Sergei Ryazansky^1^, Pavel A. Komarov^1,2^, Yuri Abramov^1^ and Alla Kalmykova^1,3^

| 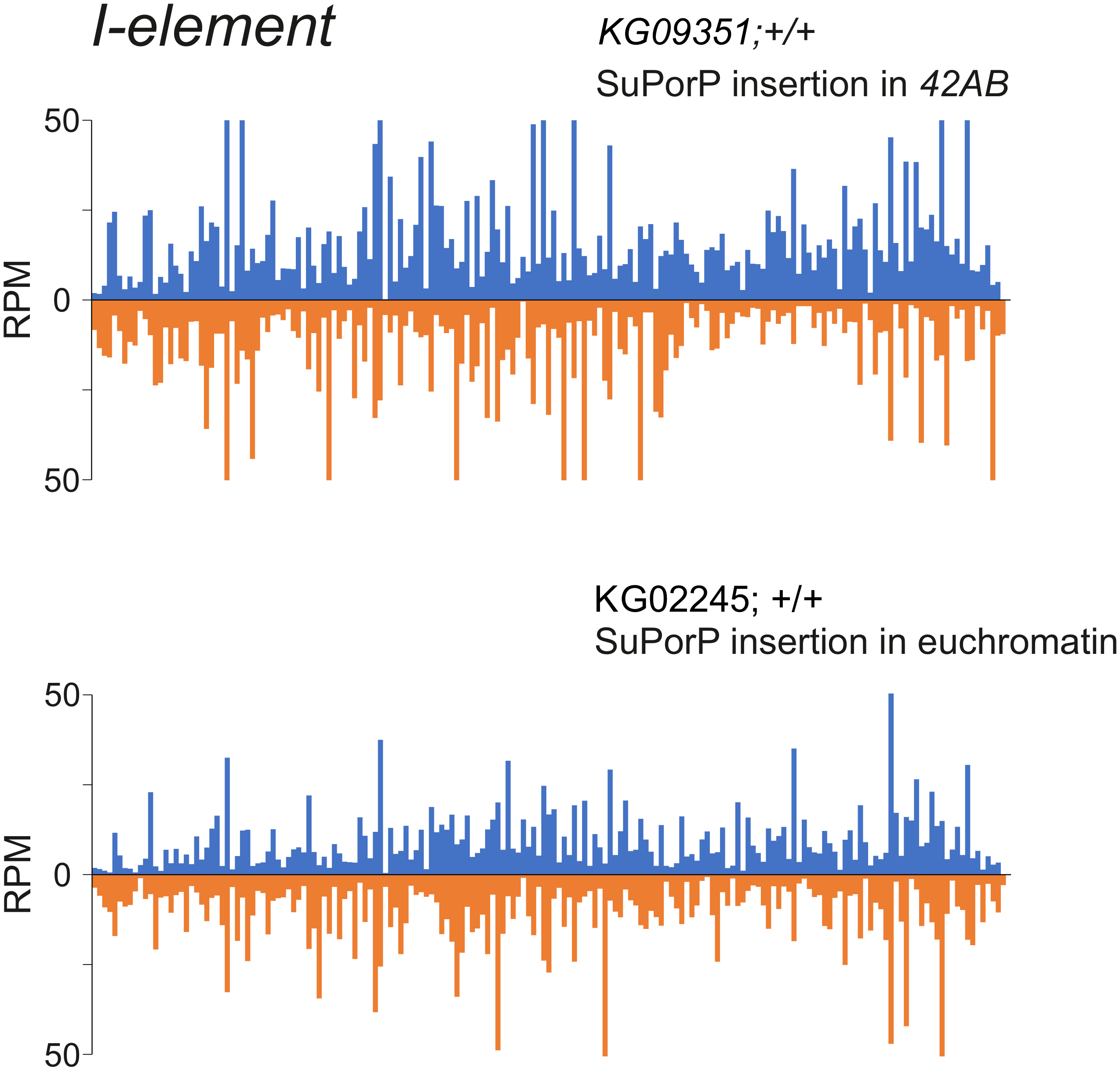 |
| --- |

**Figure S1: Small RNA mapping to the canonical *I-*element*.***  Analysis of ovarian small RNA libraries from *KG09351* (insertion in *42AB*) and *KG02245* (insertion in euchromatin) strains (0-3 mismatches allowed). Reads mapped to the sense strand are shown in blue, and antisense in brown.


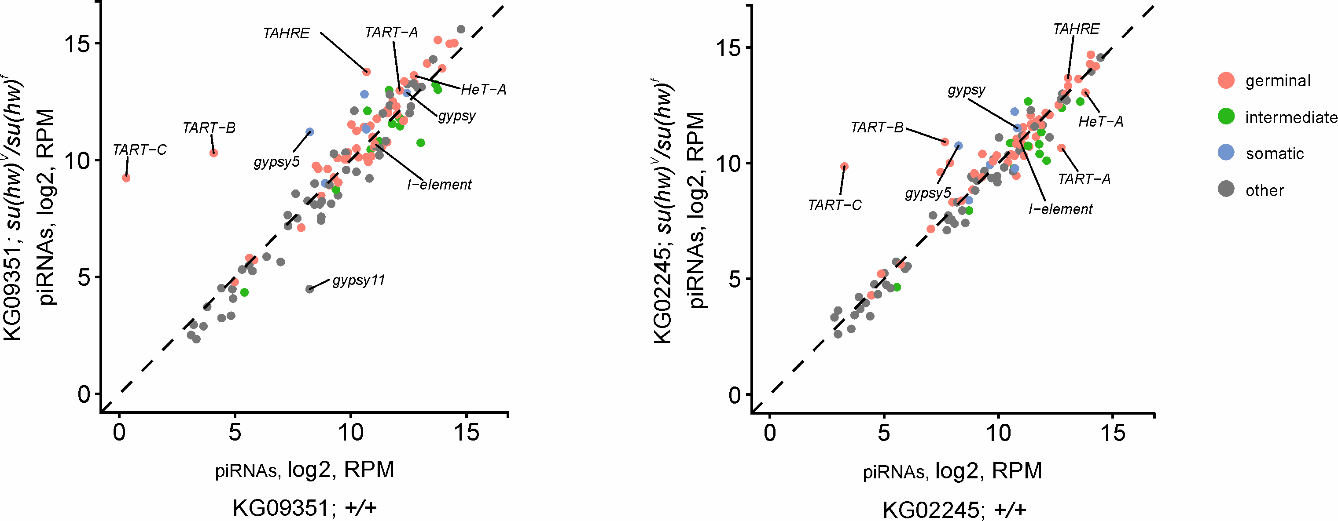


**Figure S2: Genome-wide analysis of Su(Hw) depletion on TE piRNA production (related to Figure 5).** Scatter plots of log2-transformed and RPM-normalized small RNAseq reads (24-29 nt reads are considered) in ovaries of wild type (*KG09351* and *KG02245* transgenic strains) and *su(Hw)* mutant flies (*KG09351; su(Hw)^V^/su(Hw)^f^* and *KG02245; su(Hw)^V^/su(Hw)^f^*) mapped to the canonical TE sequences.


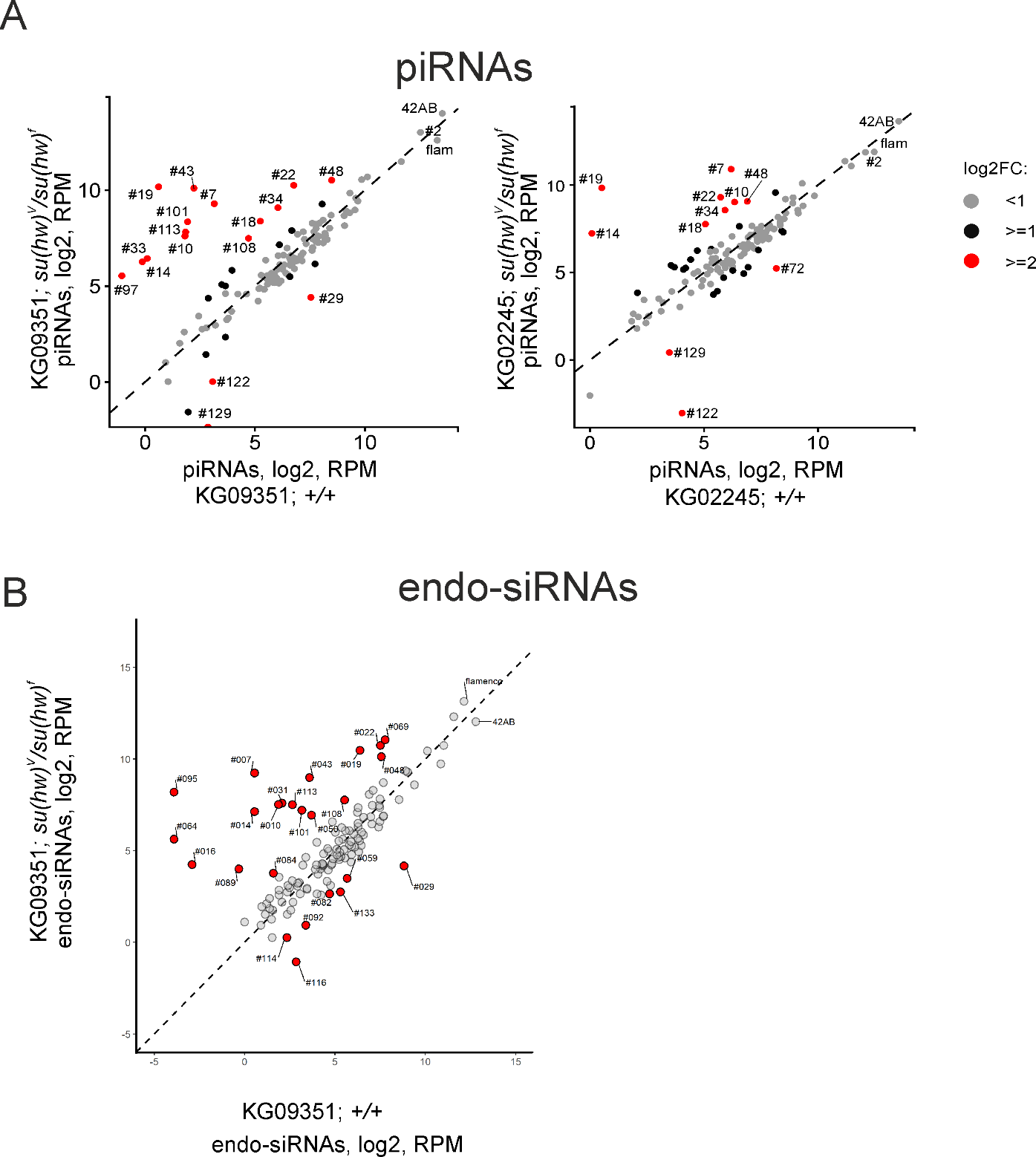


**Figure S3: Genome-wide analysis of Su(Hw) depletion on small RNA production from piRNA clusters (related to Figure 6). (A)** Scatter plot of log2-transformed and RPM-normalized small RNAseq reads (24-29-nt piRNAs were considered) in ovaries of wild type (*KG09351* and *KG02245* transgenic strains) and *su(Hw)* mutant flies (*KG09351; su(Hw)^V^/su(Hw)^f^* and *KG02245; su(Hw)^V^/su(Hw)^f^*) mapped to the master loci (ML). **(B)** Scatter plot of log2-transformed and RPM-normalized endo-siRNAs (21-nt) mapped to the master loci (ML) in the ovaries of wild type and *su(Hw)^V^/su(Hw)^f^* mutant flies. Most affected ML are related to telomeric piRNA clusters containing *TART/TAHRE* retroelements.
