# Supplementary material for: The Integrity of piRNA Clusters is Abolished by Insulators in the Drosophila Germline
Source: Genes (Basel). 2019 Mar 11;10(3):209. doi: 10.3390/genes10030209 (PMC6471301; doi:10.3390/genes10030209)
Supplement: Supplementary file 1 [file genes-10-00209-s001.zip › Table S1.docx]

**Table S1. Primers used in the study (5’-to-3’)**

| **name/target** | **orientation** | **Sequence 5’-3’** |
| --- | --- | --- |
| *rp49* | forward | ATGACCATCCGCCCAGCATAC |
|  | reverse | GCTTAGCATATCGATCCGACTGG |
| *mini-white* intron (p1) | forward | CATGATCAAGACATCTAAAGGC |
|  | reverse | AGTACCCCGAAGTATCCTAC |
| *mini-white* 1^st^ exon (p2) | forward | TGGGGTGGTGATTGGTTTTG |
|  | reverse | TTGTTCAGATGCTCGGCAG |
| *3’P-element* (p3) | forward | TAATTCAAACCCCACGGACA |
|  | reverse | ATAACATAAGGTGGTCCCGTC |
| SuPorP *gypsy* (p4) | forward | TTCTCTAAAAAGTATGCAGCACTT |
|  | reverse | CCAGTGTTTGTTCCTTGTGTAG |
| SuPorP read-through transcription (p5) | forward | AGAGGAAAGGTTGTGTGCGGAC |
|  | reverse | CCAGTGTTTGTTCCTTGTGTAG |
| *42AB*-1 (ChIP) | forward | CGTCCCAGCCTACCTAGTCA |
|  | reverse | ACTTCCCGGTGAAGACTCCT |
| *42AB*-2 (RT) | forward | TGTTTACCCAGAATGATGTTGAAATATAAGATG |
|  | reverse | CACTGACTACGGTGCCTACAGCTATG |
| *Cluster 6* | forward | ACGGGACGACTGTTTGTGCTTGG |
|  | reverse | GGCTTGTGGCTATGCTGGCGAA |
| *TART-A* | forward | AATGAACTTTGTCTGCCCTCCCA |
|  | reverse | ATCTGTCTACTGTCCGCCTTCGCTA |
| *TART-B/C* | forward | AACGACACTAATGCAACCAATG |
|  | reverse | TGTCTGTATGGGTGTGTGTCC |
| *TART-C* | forward | CACACCCACACAATATAACGACA |
|  | reverse | CTGATTCGCTTGTGAATTGC |
| *TAHRE* | forward | CATCAGACGAATCATAAACGCC |
|  | reverse | GATAAGGAGGTCATATATTAAAGGG |
| *HeT-A* | forward | CCAGGCAAGCGGACAAACGA |
|  | reverse | GGAGTGATGAGCGGCGGAAA |
| *gypsy* | forward | TTCTCTAAAAAGTATGCAGCACTT |
|  | reverse | CACGTAATAAGTGTGCGTTGA |
| *62D* | forward | TTTGGGCTTGGTGAGAACAG |
|  | reverse | TGATACCAGGCGAACAGAAATC |
| *Rab8* | forward | GAGATGGATTGGATAGCGGAC |
|  | reverse | GAACACTGGAATGACATGCTG |
